# Anorexia of Ageing, an Underappreciated Perioperative Concern?

**DOI:** 10.1002/jcsm.13683

**Published:** 2024-12-26

**Authors:** Brandon Stretton, Joshua Kovoor, Aashray Gupta, Stephen Bacchi

**Affiliations:** ^1^ Adelaide Medical School University of Adelaide Adelaide South Australia Australia; ^2^ Department of Medicine Royal Adelaide Hospital, Central Adelaide Local Health Network Adelaide South Australia Australia; ^3^ Department of Surgery Ballarat Base Hospital Ballarat Central Victoria Australia; ^4^ Department of Cardiothoracic Surgery Royal North Shore Hospital St Leonards New South Wales Australia; ^5^ Lyell McEwin Hospital Northern Adelaide Local Health Network Adelaide South Australia Australia

## Introduction

Between early 2000 and 2050, the number of people aged > 60 is expected to double, approaching 22% in developed regions [1]. As half of the population aged over 65 will require surgery at some point, reducing operative burden, particularly in the elderly, is a major public health concern [2]. One such age‐related change that was first described in 1994, which is yet to receive the recognition for its perioperative implications, is the alteration in the ability to accurately control energy intake and energy balance, ‘anorexia of ageing’ [3]. The incidence of this clinical syndrome is climbing alongside the ageing population; however, despite its high prevalence, it is often overlooked by clinicians [4, 5]. How this geriatric syndrome influences perioperative outcomes however remains unclear and requires further clarification through both clinical evaluation and research. This letter aims to provide a clear roadmap for addressing this underappreciated concern and encourage further investigation by defining the anorexia of ageing and discussing how the multifactorial aspects of anorexia of ageing may interface with the perioperative patient and their outcomes, as well as possible empiric management options.

## Definition, Pathophysiology and Diagnosis

Anorexia of ageing is a geriatric syndrome that is defined by decrease in energy intake, and the reduction in appetite that underlies it, with associated undernutrition, unintended weight loss, immunocompromise/senescence, frailty and by association, sarcopenia, functional impairment, loss of independence and quality of life, alongside other adverse health outcomes [6]. Energy intake decreases by ~30% between the ages of 20 and 80, with elderly tending to consume smaller, less frequent meals more slowly and overall, be less hungry, with more rapid satiation than young adults [7]. It is a common syndrome, affecting up to 25% of community dwellers and 85% of aged care facility clients [8]. It is characterised by a multifactorial process involving physical, societal and physiological factors.

Age‐related functional impairments (such as immobility, visual decline, poor dentition and neurocognitive impairment) can impair a person's ability to shop, prepare and consume food and are associated with poor protein intake [7]. Societal factors such as financial limitations and loneliness also contribute to reduced oral intake by precluding access to nutrition and decreasing appetite, with elderly people consuming up to 50% more in the company of friends compared to eating alone [7].

There are also physiological changes that contribute to the anorexia of ageing, with changes in homeostatic mechanisms, neurotransmitter/hormonal function and the alimentary tract. Homeostasis of energy intake is impaired in the elderly. When a caloric restriction is imposed on elderly individuals, they do not demonstrate the same compensatory increase in voluntary intake that is observed in younger individuals [3]. In the aforementioned 1994 study, a group of young and old men were underfed by ~750 cal for 21 days, and although the young adults quickly returned to normal weight during a period of ad libitum eating, no compensation and return to baseline was observed in the elderly [3].

Decline in taste, mucous production and myenteric cells of the alimentary tract also contribute. Sensory‐specific satiety promotes the tendency to shift consumption of food choices during a meal, promoting an increased and more varied caloric intake that is more, nutritionally balanced. The loss of smell and taste reduces the pleasurable experience of eating and limits sensory specific satiety, which may in turn promote a more restricted diet (in regard to both variety and caloric load) [7].

Additionally, myenteric plexus function and fundic nitric oxide concentrations deteriorate with age, which leads to a more rapid fundus dilation, slowed gastric emptying and increased fullness with earlier satiety and meal cessation [7]. The neuro‐hormonal regulation of appetite involves a complex interaction between the central feeding system (neurotransmitters) and peripheral feeding system (hormonal), which both experience age related changes. Age‐related central neurotransmitter changes that contribute to the anorexia of ageing include relative increases in serotonin and cocaine‐amphetamine‐regulated transcript, with decreases in endogenous opioids. Concomitant hormonal changes in the peripheral feeding system include increases in cholecystokinin, glucagon‐like peptide‐1 (GLP‐1), leptin and decreases in ghrelin. Additionally, the stress of age, which increased catecholamines and cortisol levels with decreased sex and growth hormones, stimulate the release of interleukin‐6 and tumour necrosis factor alpha (TNF a) which both reduce food intake.

Diagnosis of the anorexia of ageing remains heterogenous, with a variety of different assessment modalities used (Supporting Information). Although it is unclear which method is optimal, the most utilised tool to screen for this syndrome is the Mini Nutritional Assessment Short Form [6]. Despite the heterogeneity in assessment modalities, clinicians must recognise that it cannot be assess via body mass index and, instead, must specifically assess appetite [9].

## Interface and Clinical Impact on the Perioperative Patient

The clinical impact of the anorexia of ageing is a pertinent perioperative concern as patients are already impact by ‘perioperative anorexia’ as a separate entity, which is a common, ubiquitous surgical complication, occurring in over 50% of surgical and at least partially independent of the degree of surgical insult [10]. In uncomplicated joint replacements, the median time to return of appetite was 4 weeks [11]. Similar observations have additionally been made in cardiac and abdominal surgeries, with a progressive decline in appetite even 7 days post‐operation [10, 12].

It is crucial to differentiate between patients with pre‐existing anorexia of ageing and those who develop perioperative anorexia as a separate clinical entity. Although anorexia of ageing is a long‐standing age‐related condition characterised by diminished energy intake and impaired compensatory responses, perioperative anorexia is a transient but significant condition exacerbated by surgical stress, medications and inflammation. Patients with pre‐existing anorexia of ageing may experience worsening of their anorexia post‐operatively, whereas patients without anorexia of ageing are still at risk for perioperative anorexia, especially if they are frail or elderly.

With regard to perioperative anorexia, the limited literature suggests that there is a ‘dual pathology’ with reduced intake (from reduced appetite) and increased caloric expenditure. In a study of aortic valve replacement recipients, baseline energy expenditure increased by 20% from 1358 kcal to 1613 (*p* = 0.002). This increase in expenditure corresponded with a significant fall of 75% in food intake in Day 4 of the post‐operative period [13]. This study also demonstrates that pre‐ and post‐operative weight evaluation is a poor surrogate marker for energy balance as in spite of the negative energy balance; the average weight of patients increased by 2 kg. An analysis of body composition attributed the weight change to blood volume expansion (median gain of extracellular fluid = 1.1 kg), likely driven by a systemic inflammatory response syndrome, as was reflected by CRP elevations [13]. Salle et al. hypothesised that cytokines (TNFa and IL1b) involved in the systemic inflammatory response may also reduce appetite by their interaction with hormonal and neurochemical mediators regulating food intake, such as leptin, ghrelin and neuropeptide Y [13].

Although a decline in appetite is often anticipated post‐operatively, particularly in elderly patients, the failure to adequately compensate for this decline is of major concern, especially for patients with pre‐existing anorexia of ageing. In such patients, the perioperative phase poses an even greater challenge, as their compromised nutritional intake can exacerbate the frailty and functional impairments typically associated with anorexia of ageing. Chiefly, selective malnutrition that results from this anorexia complex directly compromises both morbidity and mortality [14]. Resultant sarcopenia and frailty reduce the physiological reserve for recovery after surgery, impairing wound healing and functional recovery, which, in conjunction with immunosenescence, results in an increase in healthcare resource utilisation, prolonged length of stay, nosocomial infections (independent of the effects of length of stay and comorbidity), care requirement on/location of discharge and inpatient mortality [15, 16]. Furthermore, perioperative anorexia, even in patients without prior anorexia of ageing, can lead to detrimental post‐operative outcomes such as delayed wound healing, increased risk of infection and prolonged hospital stays.

## Management Strategies

The management of anorexia of ageing is challenging, with a recent survey demonstrating unanimous agreement in this challenge, due to the lack of high‐quality evidence to guide treatment [6]. However, first principles and preliminary evidence suggest that early detection and intervention is critical if there is any chance of abrogating the consequences of anorexia of ageing, particularly in the perioperative setting [9].

At current, achieving 60% of the estimated requirements is considered acceptable according to the ESPEN guidelines, with nutritional support only being suggested when intake if < 60% for > 10 days [17]. Firstly, elderly patients should probably not be allowed to forego 40% of their estimated requirements in the post‐operative period as is currently allowed. Diets rich in carbohydrates and unsaturated fats can maximise perioperative appetite compared to those of high protein and fibre [18]. Additionally, although pleasurable, tasteful meals may promote caloric intake, the severity of caloric restriction in this setting must not be underplayed, and instead calories should be considered a medicine and need not be pleasurable. Empiric perioperative micronutrition also serves as a potential therapeutic avenue [19]. Allied health input for dentition and swallowing function assessment may also provide benefit. Input optimisation should be met with nutrient loss minimisation, ensuring diligent attention to post‐operative nausea, vomiting, diarrhoea and where appropriate, minimising perioperative fasting windows, high stoma outputs and post‐operative ileus.

There are several pharmacotherapy options available for consideration, although their use is limited by side effects and limited evidence. Commonly utilised appetite stimulants (orexigenic) include anabolic steroids, thalidomide (TNF inhibitor) and megestrol acetate (synthetic progestin) [20]. Although agents can improve appetite and quality of life, their respective risk profiles often outweigh the benefits and as such are not recommended in the long‐term management of anorexia of ageing [21, 22]. However, their utility in short, perioperative settings remains unclear and have strong potential. Alternative medications for consideration include mirtazapine (atypical antidepressant) and anamorelin hydrochloride (ghrelin receptor agonist) [23, 24]. Concurrent to medication introduction, suspending medications that may produce appetite reduction of delay in gastric motility should be considered.

## Conclusion

Perioperative anorexia, in conjunction with the anorexia of ageing is a very common clinical scenario that poses a significant threat to the elderly, is not widely appreciated and has major therapeutic implications for the perioperative period. Considering the ageing population and increasing pressures for hospital beds and rapid surgical recovery, further research into this complex interaction is warranted. Addressing both pre‐existing anorexia of ageing and perioperative anorexia requires a multifaceted approach, as both conditions can significantly influence surgical outcomes in elderly patients. Early identification and intervention strategies, including tailored nutritional support and pharmacological options, are critical. This clinical scenario, though common, remains underappreciated, and further research is warranted to refine diagnostic methods and optimise management protocols, particularly for patients undergoing major surgeries. Proceeding straight to intervention is potentially justifiable, although cognisance regarding this evidence free practice is required. Future research efforts should seek to optimise the methodology of diagnosis, the surgeries that are most likely to provoke this anorexic syndrome, timing and duration of therapeutic, supplementary or prophylactic intervention.

## Conflicts of Interest

The authors declare no conflicts of interest.

## Supporting information


**Data S1** Supporting Information.
