# Supplementary material for: Anorexia of Ageing, an Underappreciated Perioperative Concern?
Source: J Cachexia Sarcopenia Muscle. 2024 Dec 26;16(1):e13683. doi: 10.1002/jcsm.13683 (PMC11669948; doi:10.1002/jcsm.13683)
Supplement: Supplementary file 1 — Data S1 Supporting Information. [file JCSM-16-e13683-s001.docx]

Supplemental Information

# Diagnostic modalities

**Visual Analogue Scale (VAS)(1)**

VAS are most often composed of lines (of varying length) with words anchored at each end, describing the extremes (that is, `I have never been more hungry'=`I am not hungry at all'). Subjects are asked to make a mark across the line corresponding to their feelings. Quantification of the measurement is done by measuring the distance from the left end of the line to the mark


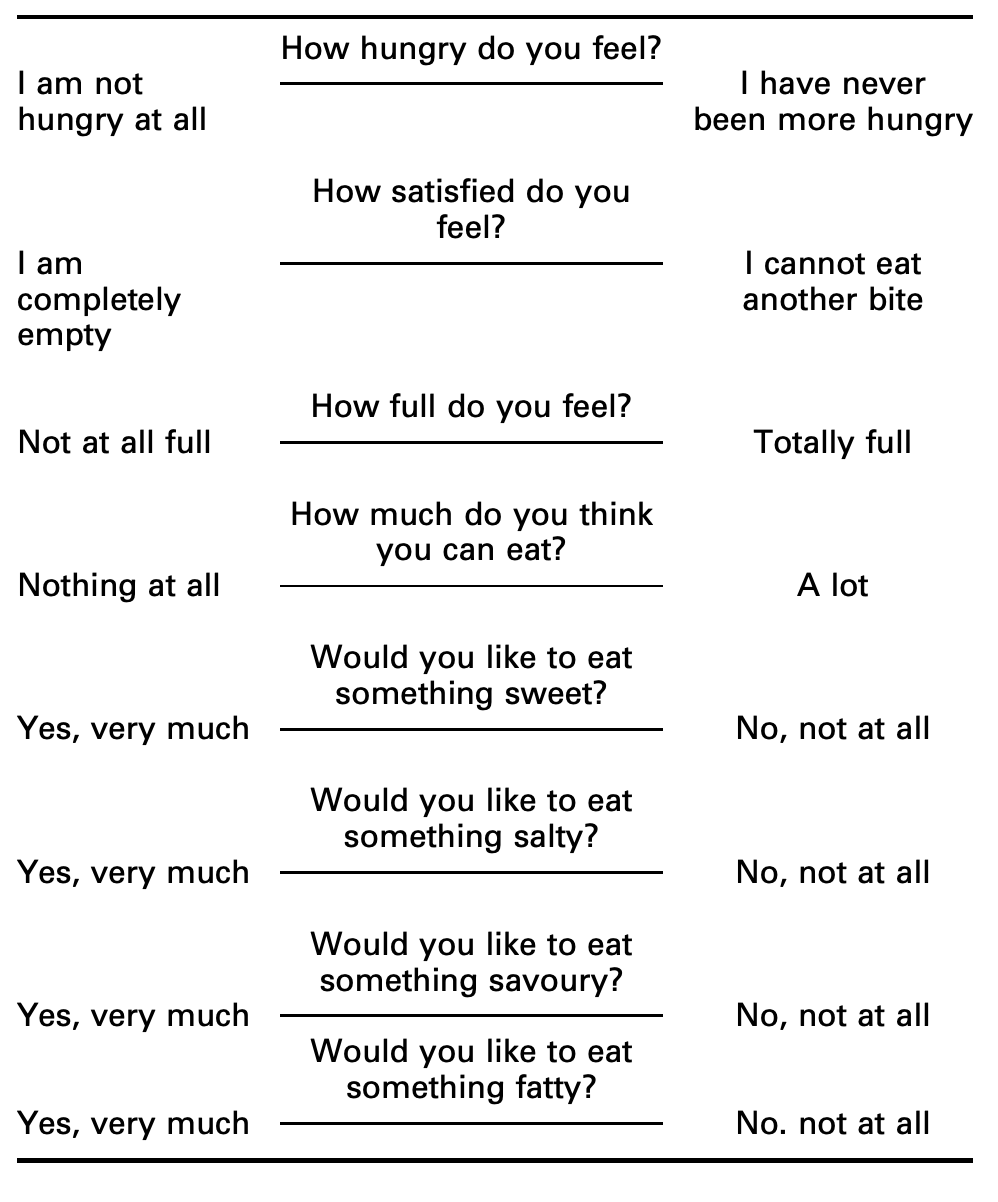


**Appetite, Hun-ger and Sensory Perception Questionnaire (AHSPQ)(2, 3)**

AHSP consists of 29 questions with diversified answers centered on the self-assessment of the energy and macronutrients intake, hunger sensation, appetite, taste, and smell both currently as well as in previous times.

The questionnaire is divided into 5 sections:

- Present Taste Perception: 8 items, range 8 to 40.
- Appetite: 6 items, range 6 to 30.
- Present Smell perception: 3 items, range 3 to 15.
- Present Smell perception compared to the past: 3 items, range 3 to 15.
- Daily feelings of Hunger: 9 items, range 9 to 45.

For each item, the person interviewed can choose among 5 possible variables, and for any answer, the score goes from 1 to 5. A low score would indicate a deterioration while a high score would indicate an adequate perception

**Council on Nutrition Appetite Questionnaire (CNAQ) and its short derivative, the Simplified Nutritional Appetite Questionnaire (SNAQ)(4)**


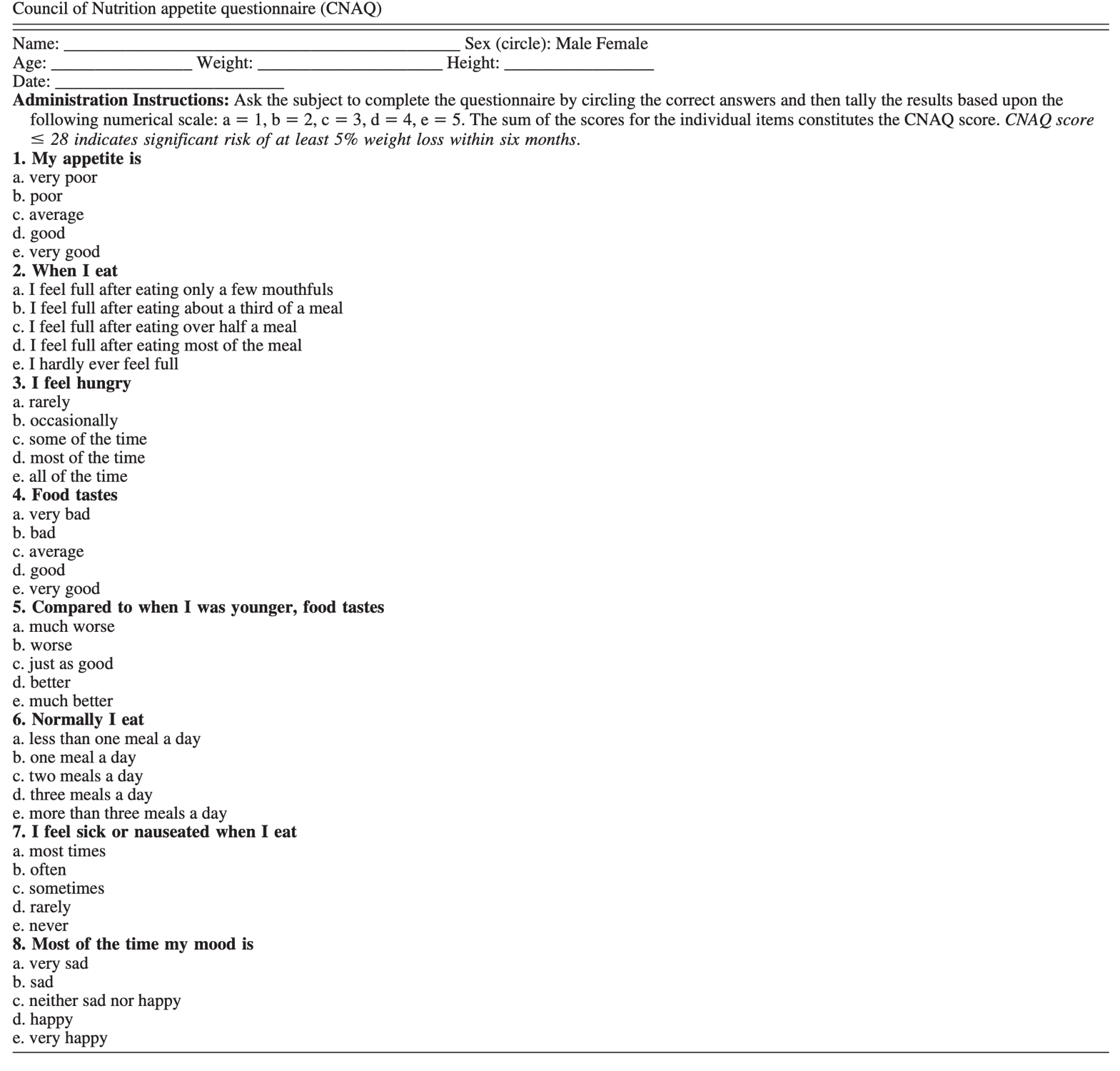


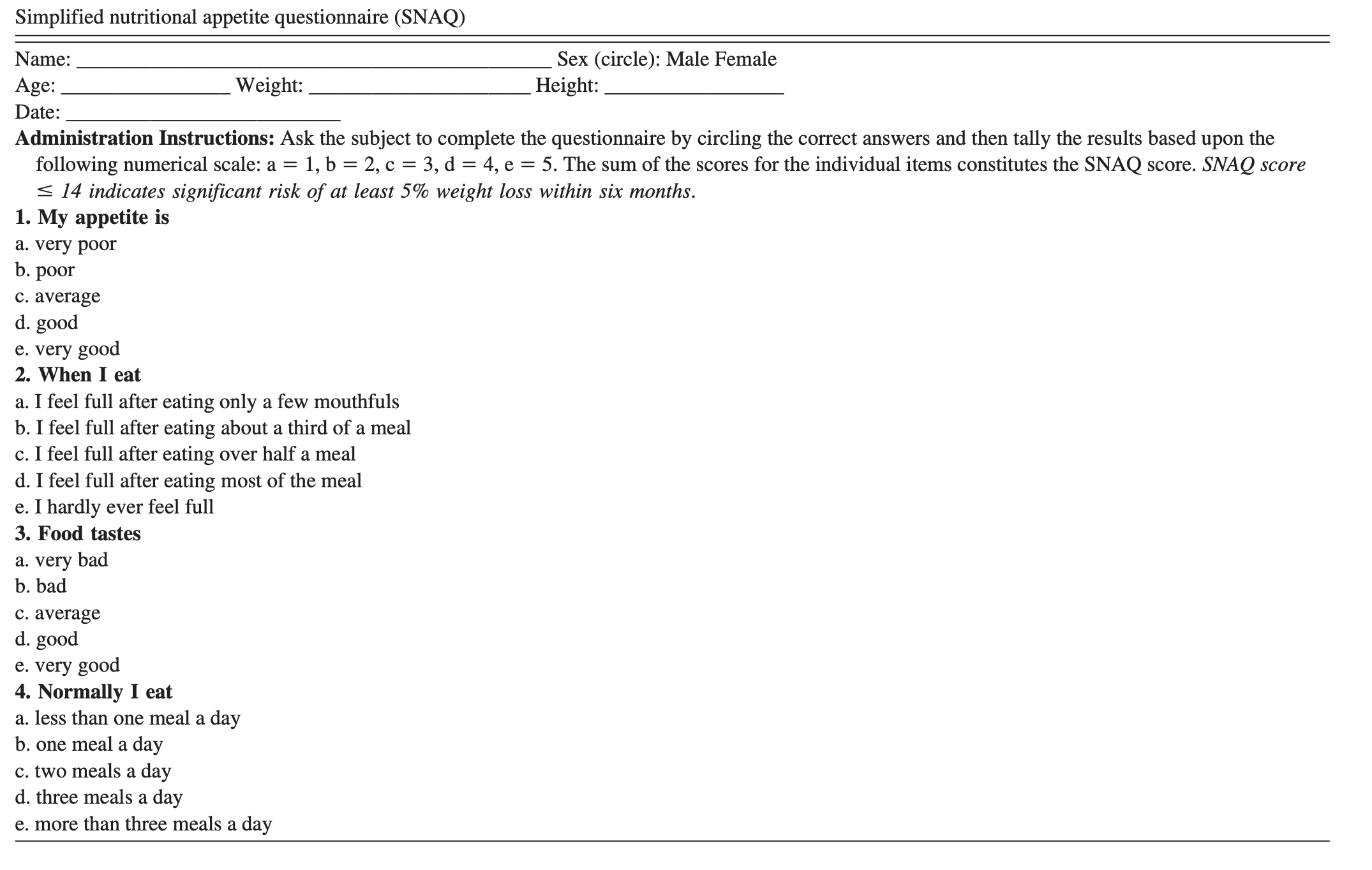


**The Functional Assessment of Anorexia and Cachexia Therapy (FAACT)(5)**


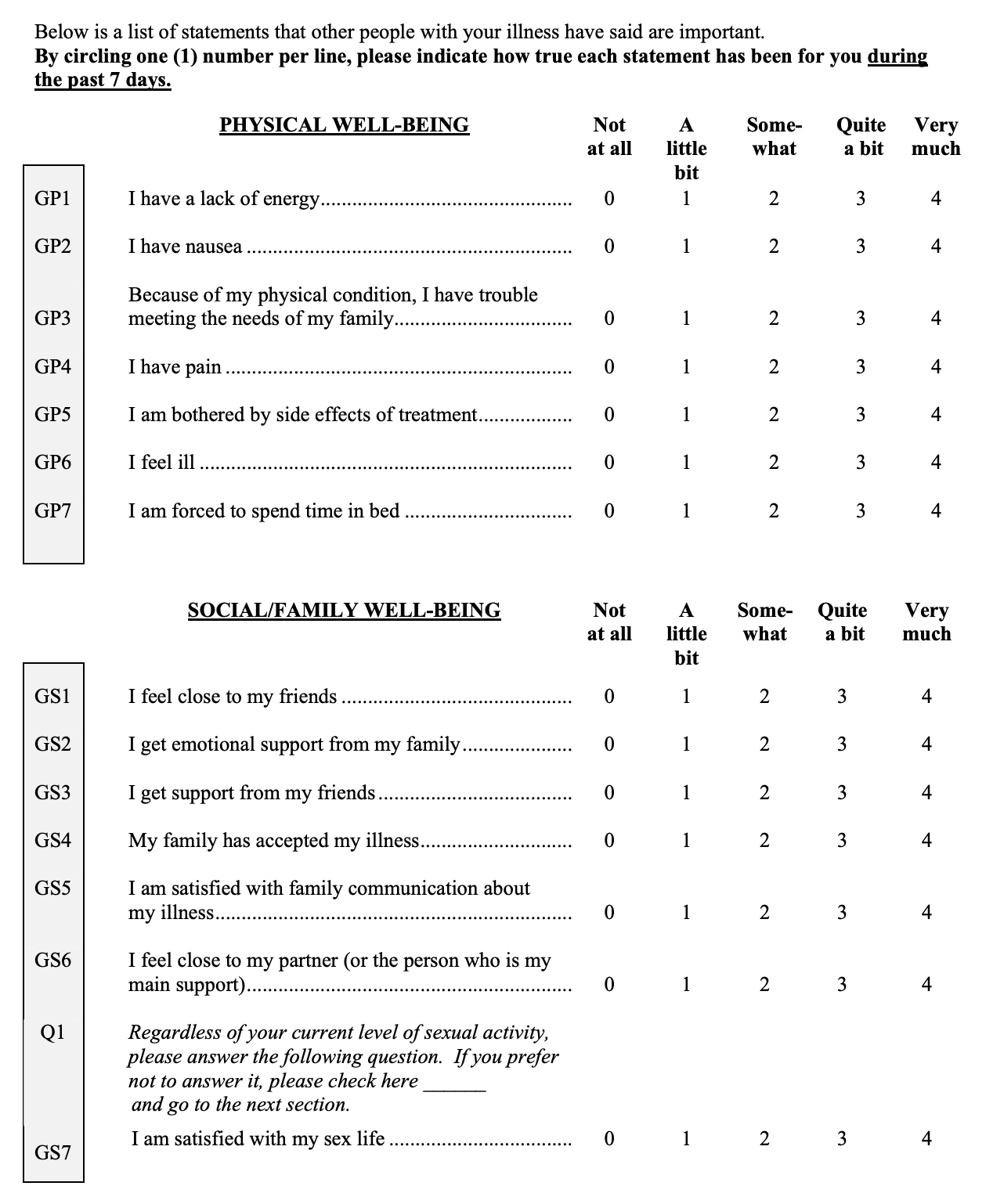


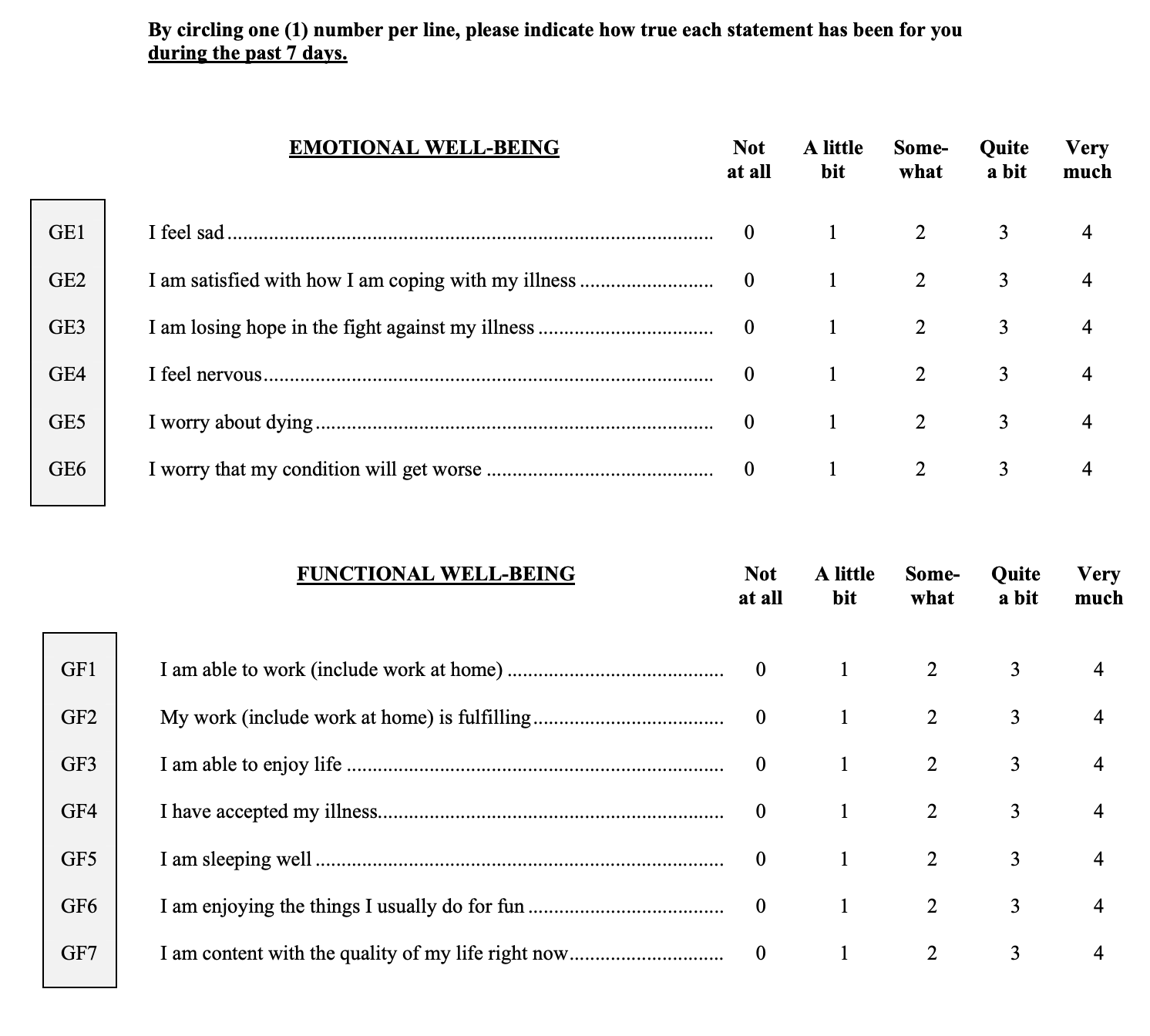


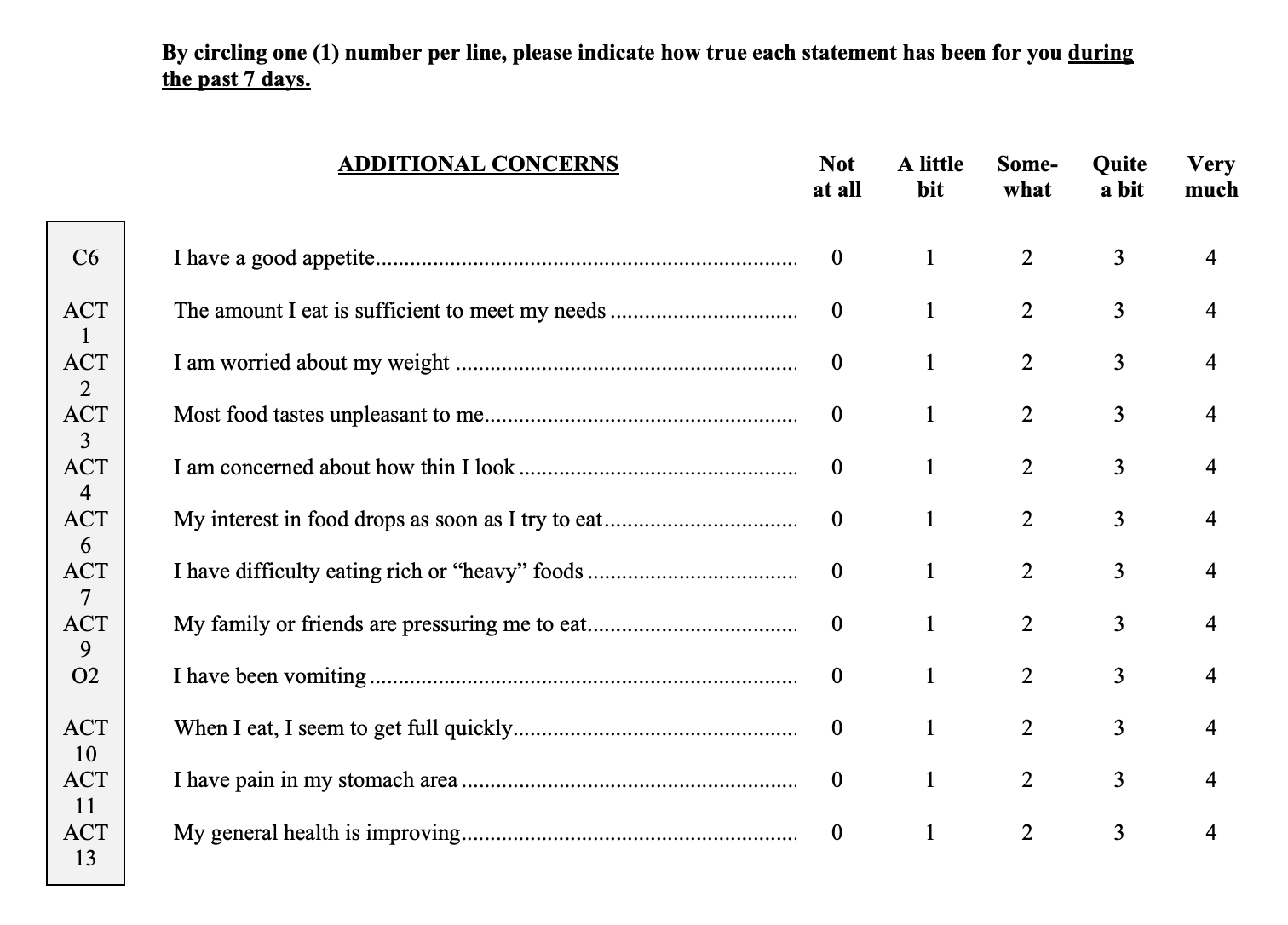


**Rapid Geriatric Assessment (RGA)(6)**


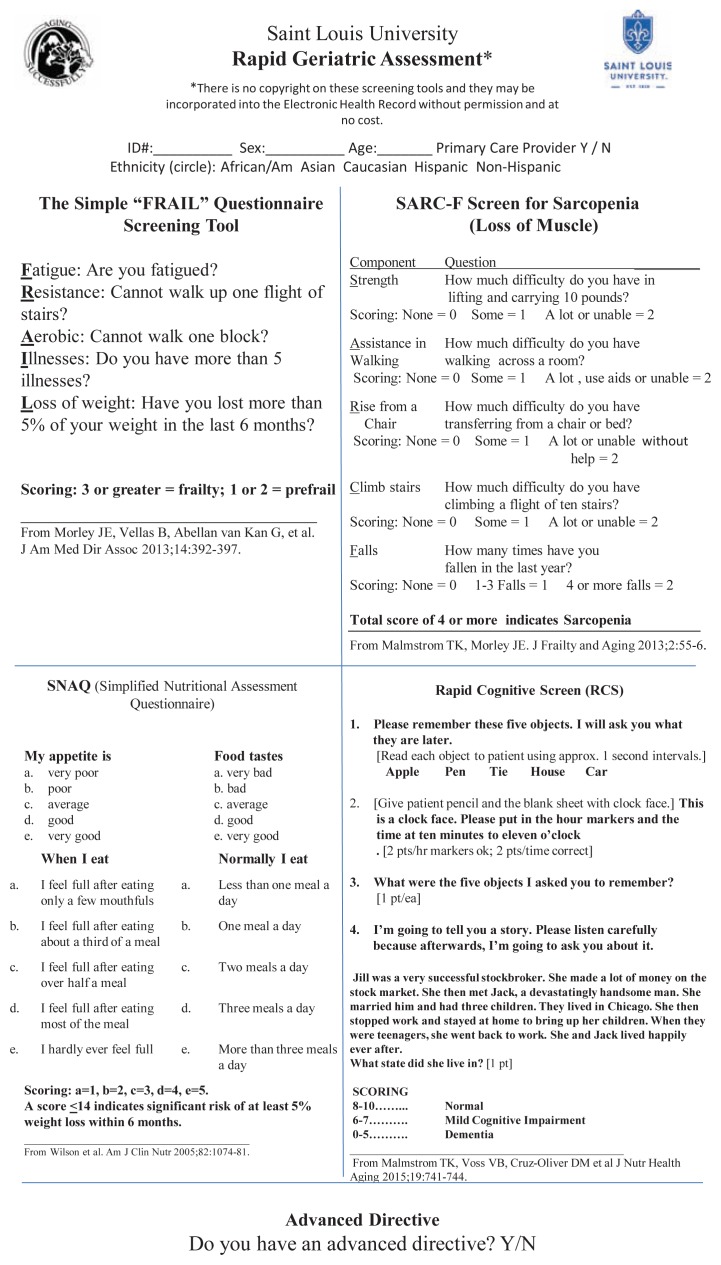


# Additional perioperative factors that influence appetite:

Additionally, the patient’s appetite in the perioperative period is also influences by factors such as anaesthesia, medications, gastrointestinal function and environment. The impact of anaesthetic agent on caloric intake in humans remains inconclusive, with variable results being demonstrated. In direct comparisons, there was no difference in the median recovery time of hunger, plasma levels of ghrelin, leptin or insulin at any time-point between propofol and sevoflurane(7). Contrastingly, other studies have demonstrated a decrease in plasma serotonin levels with propofol compared with isoflurane, which was associated with an increased appetite(8). Further, patients have reported being hungrier and thirstier after xenon (inhalant) anaesthesia when compared with total intravenous anaesthesia (propofol)(9). Additionally, patients who undergo local anaesthetic methods tend to have a better postoperative appetite than those who undergo general anaesthesia(10). Exogenous opioids in humans may have no direct effect, or be slightly anorexic(11). The potential anorexic effects of opioids must be balanced however, with postoperative pain and nausea; which are intimately related with all aspects of operative recovery, including appetite(12). Postoperative gastrointestinal dysfunction is a multifaceted and common postoperative complication, which contributes to anorexia but remains poorly defined and lacks a therapeutic target(13). Postoperative ileus is also potentially exacerbated by GLP-1 agonists. Postoperative ileus also prolongs time to discharge which in itself is associated with reduced caloric intake, and likely secondary to reduced mobility and restricted food choices. Perioperative sleep deprivation is also a common, unfortunate consequence of being an inpatient(14). The current understanding of how sleep deprivation regulates the expression of appetite is mostly phenomenological, and the specific mechanisms requires further exploration however, disruption of circadian clocks has certain effects on appetite regulation(15). Consequently, perioperative sleep deprivation likely influences caloric intake through transcriptional regulation.

Supplemental Information’s References:

1. Flint A, Raben A, Blundell JE, Astrup A. Reproducibility, power and validity of visual analogue scales in assessment of appetite sensations in single test meal studies. International Journal of Obesity and Related Disorders. 2000;24(1):38-48.

2. Savina C, Donini LM, Anzivino R, De Felice MR, De Bernardini L, Cannella C. Administering the "AHSP Questionnaire" (appetite, hunger, sensory perception) in a geriatric rehabilitation care. J Nutr Health Aging. 2003;7(6):385-9.

3. Mathey MF. Assessing appetite in Dutch elderly with the Appetite, Hunger and Sensory Perception (AHSP) questionnaire. J Nutr Health Aging. 2001;5(1):22-8.

4. Wilson MM, Thomas DR, Rubenstein LZ, Chibnall JT, Anderson S, Baxi A, et al. Appetite assessment: simple appetite questionnaire predicts weight loss in community-dwelling adults and nursing home residents. Am J Clin Nutr. 2005;82(5):1074-81.

5. Ward WL, Hahn EA, Mo F, Hernandez L, Tulsky DS, Cella D. Reliability and validity of the Functional Assessment of Cancer Therapy-Colorectal (FACT-C) quality of life instrument. Quality of Life Research. 1999;8:181-95.

6. Little MO. The Rapid Geriatric Assessment: A Quick Screen for Geriatric Syndromes. Mo Med. 2017;114(2):101-4.

7. Besnier E, Perdrix A, Gillibert A, Selim J, Froëmer B, Ghemired A, et al. Postoperative hunger after outpatient surgery in patients anesthetized with propofol vs sevoflurane: a randomized-controlled trial. Canadian Journal of Anesthesia/Journal canadien d'anesthésie. 2020;67(5):550-9.

8. Grouzmann E, Borgeat A, Fathi M, Gaillard RC, Ravussin P. Plasma and cerebrospinal fluid concentration of neuropeptide Y, serotonin, and catecholamines in patients under propofol or isoflurane anesthesia. Can J Physiol Pharmacol. 2000;78(2):100-7.

9. Coburn M, Kunitz O, Baumert JH, Hecker K, Rossaint R. Patients' self-evaluation after 4-12 weeks following xenon or propofol anaesthesia: a comparison. Eur J Anaesthesiol. 2005;22(11):870-4.

10. Nguyen TH, Ta NT, Dang AK, Nguyen TT, Dam VAT, Latkin CA, et al. A longitudinal assessment of appetite loss and nutritional care among postoperative patients in Vietnam. Front Nutr. 2023;10:1008636.

11. Wiffen PJ, Derry S, Moore RA. Impact of morphine, fentanyl, oxycodone or codeine on patient consciousness, appetite and thirst when used to treat cancer pain. Cochrane Database Syst Rev. 2014;2014(5):Cd011056.

12. Eriksson K, Årestedt K, Broström A, Wikström L. Nausea intensity as a reflector of early physical recovery after surgery. Journal of Advanced Nursing. 2019;75(5):989-99.

13. Kovoor JG, Stretton B, Jacobsen JHW, Gupta AK, Ovenden CD, Hewitt JN, et al. Gastrointestinal recovery after surgery: protocol for a systematic review. BMJ Open. 2021;11(10):e054704.

14. Kovoor JG, Stretton B, Kerr LD, Jacobsen JHW, Hewitt JN, Ovenden CD, et al. Sleep and postoperative recovery: waking up to the evidence. ANZ J Surg. 2022;92(5):953-4.

15. Liu S, Wang X, Zheng Q, Gao L, Sun Q. Sleep Deprivation and Central Appetite Regulation. Nutrients. 2022;14(24):5196.
